# Computational Model to Predict Reactivity under Ball-Milling Conditions

**DOI:** 10.1021/acs.jctc.5c00832

**Published:** 2025-07-17

**Authors:** Raúl De Armas, Manuel Temprado, Luis Manuel Frutos

**Affiliations:** a Departamento de Química Analítica, Química Física e Ingeniería Química, Grupo de Reactividad y Estructura Molecular (RESMOL), 16720 Universidad de Alcalá, Alcalá de Henares, Madrid 28801, Spain; b Universidad de Alcalá, Instituto de Investigación Química ‘‘Andrés M. del Río’’ (IQAR), Alcalá de Henares, Madrid 28801, Spain

## Abstract

A computational model to estimate the mechanical work of activation for a chemical reaction under ball-milling conditions is developed. The model uses a simple force scheme based on isotropic compression (“wall-type forces”) to mimic the effect of ball collisions. It calculates the mechanical work applied along the reaction path and predicts the variation of the activation energy. The forces are applied in all possible directions to simulate the random nature of the impacts. The model is tested on different systems including reactions with known experimental mechanochemical behavior. The model was applied to two representative Diels–Alder systems and [2 + 2] cycloaddition to test its predictive capacity. The model predictions agree with the main experimental trends and confirm that mechanical forces play a significant role in controlling the reactivity. The results bring to light the importance of mechanical work in driving selectivity under ball-milling conditions and demonstrate that such forces can differentially affect the forward and reverse directions of a chemical equilibrium. The model is simple to implement and permits the identification of whether a reaction is likely to be promoted by ball milling.

## Graphical abstract

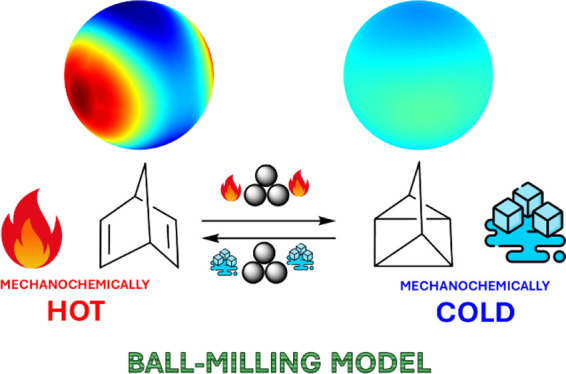

## Introduction

Mechanochemistry involves chemical reactions initiated or accelerated by mechanical energy, such as compression, shear, or friction. Application of mechanical forces can be done in an atomic-level controlled way,^1^ conforming the so-called directed mechanochemistry, or through macroscale, isotropic methods such as ball milling, where repeated impacts and shear between milling media and powders generate the conditions necessary for chemical transformation.^2^

In directed mechanochemistry, the mechanical forces are applied in a very controlled way (usually, the forces are applied to specific atoms and the force magnitude could also be precisely controlled), inducing specific changes in molecular structures,^3^ leading to bond formation or cleavage.^4^ On the contrary, bulk mechanochemistry focuses on processes like grinding or milling, where mechanical actions are not controlled at the molecular level; nevertheless, these forces may also induce chemical transformation of reacting materials. Ball milling is a relevant technique in mechanochemistry, utilizing mechanical energy to facilitate chemical reactions and is employed not only in industry but also in lab operations.^5^ This method involves grinding reactants together with milling balls in a rotating or vibrating chamber, promoting reactions through mechanical impacts. Ball milling has been employed in various applications, including the synthesis of complex compounds,^6–8^ the preparation of nanomaterials,^9,10^ and the development of single-atom catalysts.^11–13^

In these cases, all reagents are mixed typically in the solid state and in the absence of solvents, significantly reducing waste and offering a more sustainable and environmentally friendly alternative to traditional solution-based synthesis.^14^ Furthermore, one of the most promising applications of ball milling is its ability to enhance or switch reaction selectivity as compared with conventional liquid-phase-based reactions. Under conventional solution-phase conditions, competing reaction pathways often lead to product mixtures. In contrast, mechanochemical conditions such as those in ball-milling reactors can alter reaction pathways and favor the formation of a specific product, sometimes distinct from that obtained via thermal activation in solution.^2,15–17^

Modeling mechanochemical processes computationally is inherently challenging due to the diversity and complexity of the mechanical forces involved. Nevertheless, simple theoretical frameworks, such as Bell’s model,^18^ can provide a first approximation to how mechanical forces modify reaction energies or activation barriers. Beyond these, more advanced models and computational tools have been developed. These include different theoretical approaches^1^ and quantum chemical methods, such as force analysis techniques,^19^ that allow a more detailed and accurate description of how mechanical forces influence chemical reactivity.

Although most of the theoretical and computational models have been developed for ground-state mechanochemistry, there are also specific models and computational protocols to study the mechanical control of photochemical,^20–23^ and photophysical processes.^24,25^

Bulk mechanochemistry is even more complex to study from a computational point of view due to the complexity of the experimental setups. Despite these challenges, several theoretical and computational approaches have been developed to model mechanochemical processes under ball-milling conditions.

DFT calculations with microkinetic modeling have been used to reproduce experimentally observed reaction times for different chemical processes under ball-milling conditions, where the mechanochemical acceleration is explained without invoking specific force–reactant interactions. Instead, the key mechanistic effect was attributed to a high local concentration of reagents and a distinct effective dielectric constant: replacing the solvent medium (or vacuum) with a dielectric constant representative of the mixed solid reactants. This strategy led to significantly different computed activation barriers and reaction rates.^16,26^ Additionally, it has been proposed that mechanical forces can alter the curvature of the potential energy surface at the transition state, particularly when the applied forces are orthogonal to the reaction coordinate.^27^

However, to date, no mechanochemical models have been developed permitting the quantification of the mechanical work exerted during ball milling and its effect on the activation energy of a chemical reaction.

Here, we propose a simple mechanochemical model to account for activation energy variation in ball-milling reactions. This model permits the quantification of the viability of a mechanochemical reaction induced by ball milling, quantifying also the amount of mechanical energy that can be employed to facilitate a chemical process. We apply it to some simple systems in order to provide practical insight into the application of the method as well as to test its reliability.

## Methodologies

The developed method to compute the mechanical work due to ball milling is based on knowledge of the molecular structure for minimum and transition states for a given chemical reaction. We have implemented this model in the fashion described below in Python and is available in a public repository.^28^

The different molecular structures for the case study employed in this work are obtained in the following way: the minima and transition state structures for the case of the norbornadiene/quadricyclane system have been taken from a complete active space self-consistent field (CASSCF) with a (4,4) active space, using a 6-31G­(d) basis set.^21^ In the case of Diels–Alder reactions involving DMA/MA, MA9/BQ, and H9/BQ, the corresponding structures were determined by Pladevall et al. using the B3LYP-D3 functional with a 6-311++G­(d,p) basis set.^26^

Finally, for study of the Diels–Alder reaction between diphenylfulvene and maleimide, we employed the transition state structures determined by Sakai et al.^27^ being the reactants those minima directly linked to the transition states in all cases. The structures were determined at the B3LYP/6-311G­(d,p) level of theory.

## Results

A mechanochemical model will be developed to simulate the ball-milling processes. The model is based on a microscopic description of the milling event. The types of forces applied are wall-type forces, and they are applied isotropically (see Figure 1).

**Figure 1:**
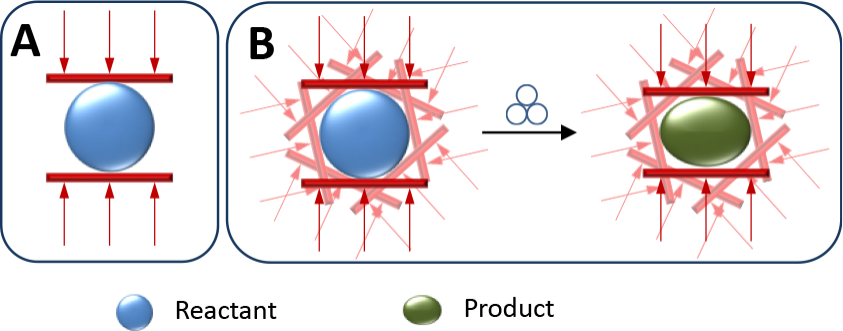
(A) Schematic representation of the effect of an individual collision of milling balls on a reagent. Two walls generate a compressive force on the reagent. (B) When all directions (statistically equiprobable) are considered, the effect of random collisions on the mechanochemically induced reactivity of the reagent can be determined to yield the product.

In addition, during the impact time, these forces can perform work that may reduce the activation barrier of the chemical process under consideration. This effective mechanical work can be determined in all spatial directions and added to define the activation energy variation under ball-milling conditions.

### Wall Force Algorithm for Ball Milling

The proposed theoretical model predicts the variation in the activation energy for a given reaction pathway due to the milling effect and is based on the following assumptions:

1) The reactants are already prepared for reaction, i.e., the mixture of reactants (adduct in the case of bimolecular reactions) is available to some extent.
2) The potential reaction pathways of the system are not altered by the application of the force provided by the ball’s collisions.
3) The forces exerted by the balls in a collision event can be considered as two-wall compression forces. This assumption relies on the fact that the ball radius is much larger than reactant’s size.
4) The magnitude of the applied forces to the reacting system is derived from the average pressure reached in the ball’s impact events and is considered constant along the reaction path.

It is necessary to discuss the limitations of these conditions and how they affect the prediction of the mechanochemistry.

Consideration (1) implies that a mixture of reactants is reached, to some extent, before proceeding with a mechanochemical reaction. Of course, the milling process itself could contribute to mix the reactants, but in any case, we assume that some fraction of the reactants are ready to react mechanochemically.

Regarding condition (2), this assumption means that the main features of the potential energy surface (PES) (such as local minima, transition states, and reaction paths) do not change when forces are applied. In other words, mechanochemical activation does not create new reaction pathways or remove existing ones nor modifies the structure of minima or transition states. The mechanical force has the only effect to develop a work favoring or disfavoring the reaction. Therefore, it permits to treat mechanical force as a factor that may change the feasibility of a reaction pathway but not to alter it.

This approach has a limitation since it does not include cases where forces change the PES in a more fundamental way. For example, strong forces may provoke the appearance of new reaction intermediates or even new transition states that may eventually be accessible in mechanochemical conditions and induce new reactivity. This model is unable to deal with these cases.

Assumption (3) is reasonable, but it simplifies how forces are transmitted by using a two-wall force model. In real systems, ball collisions are more chaotic, the direction of the force changes, and shear forces can appear, for instance. By treating the force as a uniform compression between two infinite walls, a simplified picture of the real forces is considered, making it relatively easy to compute the developed work and its effect on the energetics of the reaction.

Finally, condition (4) basically assumes a constant mechanical force during the mechanochemical reaction. This approach is reasonable as the reaction time is shorter than the impact time, which implies that the reaction takes place only by mechanical activation.

Of course, this model does not account for the eventual catalytic effect of the ball itself,^29^ and it only describes the mechanical effect (i.e., work) developed by the balls.

The model presented here is designed to mimic the isotropic compressive forces characteristic of ball-milling conditions and does not rely on selecting specific pulling directions or anchoring atoms such as the widely used CoGEF (constrained geometries simulate external force) method.^30^

Based on these premises, the details of the model and its implementation is described:

#### Reaction Path and Activation Energy

The model starts from a known reaction pathway obtained from quantum chemical calculations. The structure of the reactant is defined by coordinate vector **Q**_min_, which corresponds to a local minimum on the potential energy surface (PES). The transition state is defined by vector **Q**_TS_, which is a first-order saddle point on the same surface. The activation energy in the absence of external forces is given by the energy difference between these two stationary points:

$$E_{a}=E\left(Q_{\mathrm{TS}}\right)-E\left(Q_{\min}\right)$$ 1

The molecular system used in the model includes only the reactive part of the chemical process. For unimolecular reactions, this is a single molecule undergoing transformation. For bimolecular reactions, the system includes two molecules placed in the proper reactive orientation. This reactive molecular unit is the object of all of the calculations in the model. Atomic positions are described by Cartesian coordinates, and internal degrees of freedom are considered during the evaluation of mechanical effects on the activation barrier.

The reaction pathway vector is a fundamental magnitude permitting determination of the work developed by mechanical forces, and it is defined as

$$\Delta Q=Q_{\mathrm{TS}}-Q_{\min}$$ 2

To make the two structures match as much as possible, it is necessary to eliminate translations and rotations. To do this, the two geometries were first translated so that their centers of mass coincide at the origin. The transition state structure is then optimally superimposed on the minimum-energy structure by a mass-weighted Kabsch alignment, which is rigorously free of overall translation and rotation and contains only the intrinsic structural deformation between the two configurations.^31^

#### Definition of the Reference Molecular Sphere

To define the direction and magnitude of the mechanical forces, the reactive molecular unit is first placed in a standard orientation. This means fixing a coordinate system where all atoms have well-defined Cartesian positions, **r** *_i_*, with the origin typically set near the center of mass or at a geometrically convenient point. The system is then enclosed in a virtual sphere used to define both the spatial region of mechanical action and the direction of the applied forces. The radius of the sphere, denoted as “*R*”, is defined as the maximum distance from the origin (i.e., the mass center) to any atom in the system, plus an optional correction term δ to account for atomic size or safety margin. Here, the most common way to implement the atomic size is to define δ as the van der Waals radii:

$$R=\max_{i}\left(∥r_{i}∥+\delta_{i}\right)$$ 3

This sphere serves two purposes: it provides an upper bound for the region where atoms can interact with external mechanical forces and it defines the cross-sectional area used to estimate the magnitude of such forces (*A* = π*R* ^2^). The cross section permits to determine the magnitude of the applied forces at the molecular level since the ball-milling impact pressure is an experimental parameter (usually in the range of few GPa units).

#### Directional Partitioning of the Molecular System

To simulate the directionality of mechanical impacts, a unit vector **n̂**(θ, ϕ) is defined using spherical coordinates, where θ is the polar and ϕ is the azimuthal angle. This vector represents the direction along which external compressive forces will be applied. A plane perpendicular to **n̂** passing through the origin is used to divide the system into two hemispheres. Each atom is assigned to one of the two hemispheres, depending on the sign of the scalar product **r** *_i_* ·**n̂**. Atoms for which this product is positive are located in the “forward” hemisphere, in the same direction as **n̂**, while atoms with a negative value belong to the “backward” hemisphere. This spatial division is essential to define the direction of the forces acting on each atom and to construct the “wall force” model that mimics molecular compression due to mechanical collisions.

#### Definition and Magnitude of Wall Forces

Mechanical forces are introduced through a simplified model based on directional compression. For each direction, **n̂**(θ, ϕ), atoms in the system experience forces mimicking the effect of two rigid walls compressing the molecule from opposite sides. Atoms located in the forward hemisphere (where **r** *_i_* ·**n̂** ≥ 0) receive forces in the opposite direction, −**n̂**, while atoms in the backward hemisphere (**r** *_i_* ·**n̂** < 0) receive forces in the direction **n̂**. In both cases, the forces act to compress the system along the selected direction. The total magnitude of the applied force is determined from the pressure *P* and the effective cross-sectional area *R*(θ, ϕ), approximated as the area of a circle with radius *R*, leading to a total mechanical force:

$$F_{\mathrm{mec}}\left(\theta ,\phi \right)=P\cdot \pi {\left[R\left(\theta ,\varphi \right)\right]}^{2}$$ 4

The mechanical force is a function of the direction of the applied forces since the cross-sectional area is not strictly constant for any direction (θ, ϕ). This refinement permits to take into account the nonspherical shape of the reacting system.

This total force is distributed among the atoms using weighting factors α_*i*_, which can be uniform or adjusted based on atomic properties. The force on atom *i* is then given by

$$F_{i}=\alpha_{i}\cdot F_{\mathrm{total}}\cdot {\mathrm{n̂}}_{i}$$ 5

where **n̂** *_i_* = ±**n̂**, depending on the atom’s hemisphere. These “wall forces” simulate the localized impact of mechanical compression typical of ball-milling processes (Figure 2).

**Figure 2:**
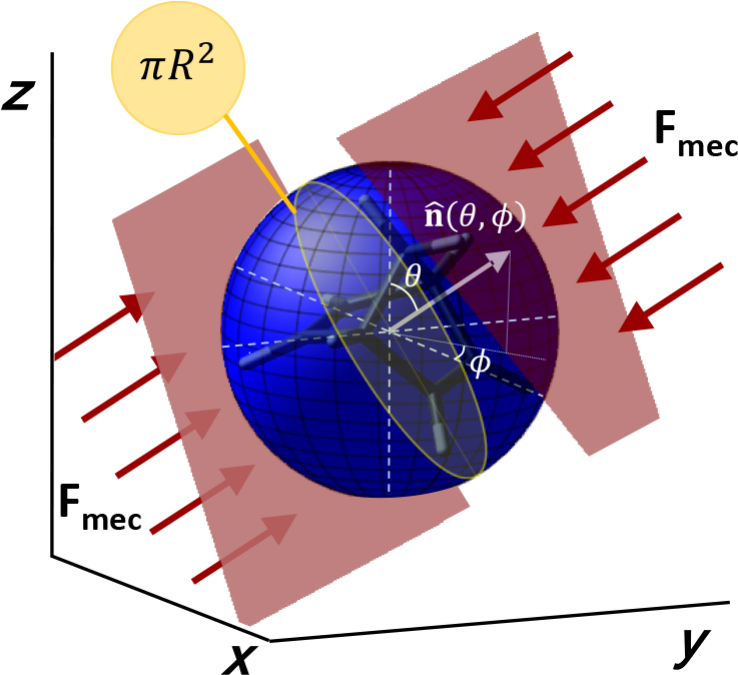
Schematic representation of the applied forces along a specific direction given by the normal vector **n̂**(θ, ϕ) where (θ, ϕ) is the spherical coordinates. The cross-sectional area of the molecular sphere is equal to a π*R* ^2^.

In the current implementation we have chosen the simplest case where all the α_*i*_ are identical, and therefore, $\alpha_{i}=\frac{1}{N}$ with *N* the number of atoms.

It is worth noting that the present model is designed to be applied on localized reactive units, such as individual mechanophores, rather than on entire macromolecular systems. In the case of larger species like polymers, the chemically active region, defined by the reactant and transition state structures connected via an intrinsic reaction coordinate (IRC), is treated independently, while the rest of the macromolecule is assumed to act as a passive spectator as its structure is usually not affected along the IRC. Although the current implementation distributes the mechanical force equally among atoms, more sophisticated schemes, such as distance-based weighting or selective application to atoms near the “impact surface,” could be introduced for larger or anisotropic systems. These refinements, while beyond the scope of the present work, represent an aspect for future development.

#### Mechanical Work and Modified Activation Energy

Once the external forces are defined, their effect on the reaction barrier is estimated through the mechanical work they perform along the reaction coordinate. The mechanical work, *W*_mec_, is calculated as the line integral of the applied forces along the reaction path from minimum geometry **Q**_min_ to transition state **Q**_TS_:

$$W_{\mathrm{mec}}=\int_{Q_{\min}}^{Q_{\mathrm{TS}}}\sum_{i}F_{i}\cdot dr_{i}$$ 6

This expression accounts for the contribution of each atomic force **F** *_i_* projected along the displacement of atom *i* along the path. In practice, this quantity can be estimated numerically by comparing the energy of the system at the minimum and transition state geometries under the action of external forces. It has to be noted that in this case, the integral expression is just

$$W_{\mathrm{mec}}=\sum_{i}F_{i}\cdot \Delta r_{i}$$ 7

where Δ**r** *_i_* = {**Q**_TS_}*_i_* – {**Q**_min_}*_i_*, where it is also necessary to remove translations and rotations in the computation of the displacement vector: Δ**Q** = **Q**_TS_ – **Q**_min_.

The mechanically induced activation energy variation is defined as the variation of the activation energy due to the mechanical action of the applied mechanical forces:

$$\Delta E_{a,\mathrm{mec}}=-W_{\mathrm{mec}}$$ 8

If *W*_mec_ > 0, then the mechanical force lowers the barrier and may enhance the reaction rate. Conversely, a negative value of *W*_mec_ indicates that the force opposes the reaction progress. Of course, once the Δ*E*_a,mec_ is determined, the corrected activation energy due to mechanical action, *E*_a,mec_, can be computed as *E*_a,mec_ = *E*_a_ + Δ*E*_a,mec_, where *E*_a_ is the activation energy in the absence of mechanical forces.

#### Averaging Forces over All Angular Directions

To account for the stochastic nature of collisions in ball milling, the effect of mechanical forces is evaluated over a range of directions. This is done by sampling different values of angles θ and ϕ, which define the direction vector **n̂**(θ, ϕ) in spherical coordinates. For each direction, the corresponding wall forces are computed, and the mechanically induced activation energy variation Δ*E*_a,mec_(θ, ϕ) is evaluated, as described previously. A numerical average over all directions provides an estimate of the overall mechanical effect on the reaction barrier. The average is calculated using a discretized form of the surface integral over the unit sphere:

$$\left\langle \Delta E_{a,\mathrm{mec}}\right\rangle =\frac{1}{4\pi }\sum_{\theta ,\varphi }\Delta E_{a,\mathrm{mec}}\left(\theta ,\varphi \right)\cdot \sin\theta \cdot \Delta \theta \cdot \Delta \varphi$$ 9

This expression includes the appropriate weight sin θ for integration in spherical coordinates. The angular resolution is determined by the step sizes, Δθ and Δϕ. This directional averaging provides a more realistic estimate of the reaction conditions under repeated mechanical impacts from random orientations. In the present implementation, the averaging over directions is performed using a uniform angular grid based on spherical coordinates. Specifically, we define “N” discrete values for the polar angle θ (ranging from 0 to π) and “2N” values for the azimuthal angle φ (ranging from 0 to 2π), resulting in a total of 2*N* ^2^ directions sampled over the unit sphere that define the numerical integration (eq 9). This choice ensures equal angular steps in both coordinates. We find that the results converge rapidly with increasing *N*; a value of *N* = 100 provides stable results while maintaining a low computational cost (see the Supporting Information for details).

#### Summary of the Model and Its Algorithm

The proposed model and its algorithm provide a mechanochemical model to estimate the effect of the mechanical impact on the activation barrier of a known chemical reaction. The input data used by the algorithm include the geometries of the reactant and transition state and a user-defined pressure *P*, mimicking the experimental setup. For each direction (θ, ϕ) given by the grid parameter *N* (see above), the algorithm defines a pair of wall forces acting on the molecule along the direction vector **n̂**(θ, ϕ), with the total force determined by the product *P*·π­[*R*(θ, ϕ)]^2^ (the van der Waals radii can be included or not in the definition of *R*). These forces are applied based on the spatial position of each atom with respect to the dividing plane orthogonal to **n̂**. The mechanical work *W*_mec_ is then calculated along the reaction path, and the modified activation energy *E*_a,mec_ = *E*_a_ – *W*_mec_ is obtained (see Figure 3).

**Figure 3:**
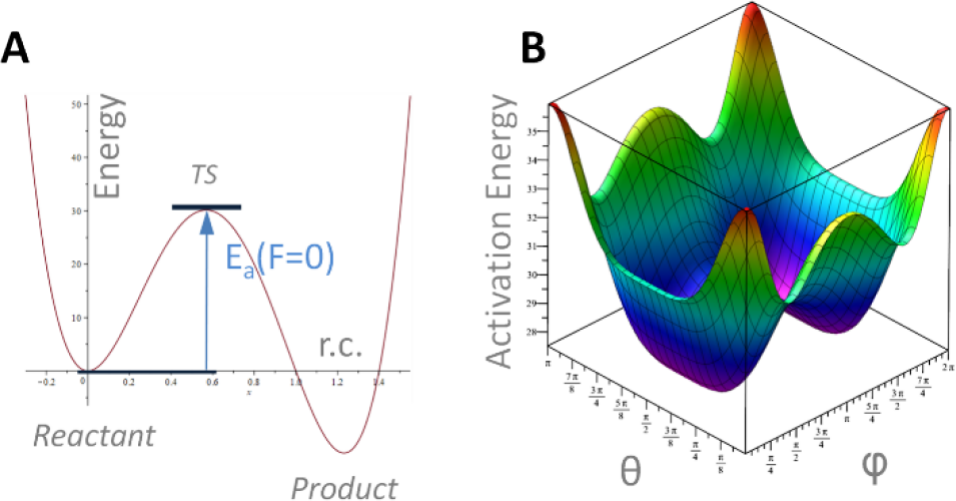
(A) Schematic profile of a chemical reaction in the absence of any mechanical force, where activation energy is defined. (B) When considering the work developed by the ball impacts (in all spatial directions), the activation energy may be affected. In general, it is a function of θ and φ.

By repeating this process over many directions, the model computes an average mechanochemical activation energy difference, ⟨Δ*E*_a,mec_⟩, which represents the effective barrier under random mechanical perturbations.

This method offers a simplified but physically grounded approach to describe how directional compression, as observed in ball milling, may influence the chemical reactivity. The model captures the anisotropic nature of collisions and provides insight into the most favorable orientations for reactivity. It can be used to screen different reactions or molecular systems and predict whether a mechanical activation pathway is viable. More advanced implementations may include direction-dependent atomic weighting, higher-resolution angular sampling, or coupling with electronic structure calculations for improved accuracy.

### Case Study

Different chemical reactions have been studied with the proposed algorithm to test the predictive capacity of the procedure. As discussed, this mechanochemical model only accounts for the mechanical effect (useful work developed by ball milling impacts promoting a given chemical reaction). Therefore, as the model cannot cover the experimental details of the setup, it is especially useful for qualitative prediction of activation energy variations as well as for differential comparison between different reaction pathways. The chosen examples cover different examples in ball-milling reactivity: unimolecular, comparative bimolecular reactivity, and selectivity for a specific bimolecular reaction (see Figure 4).

**Figure 4:**
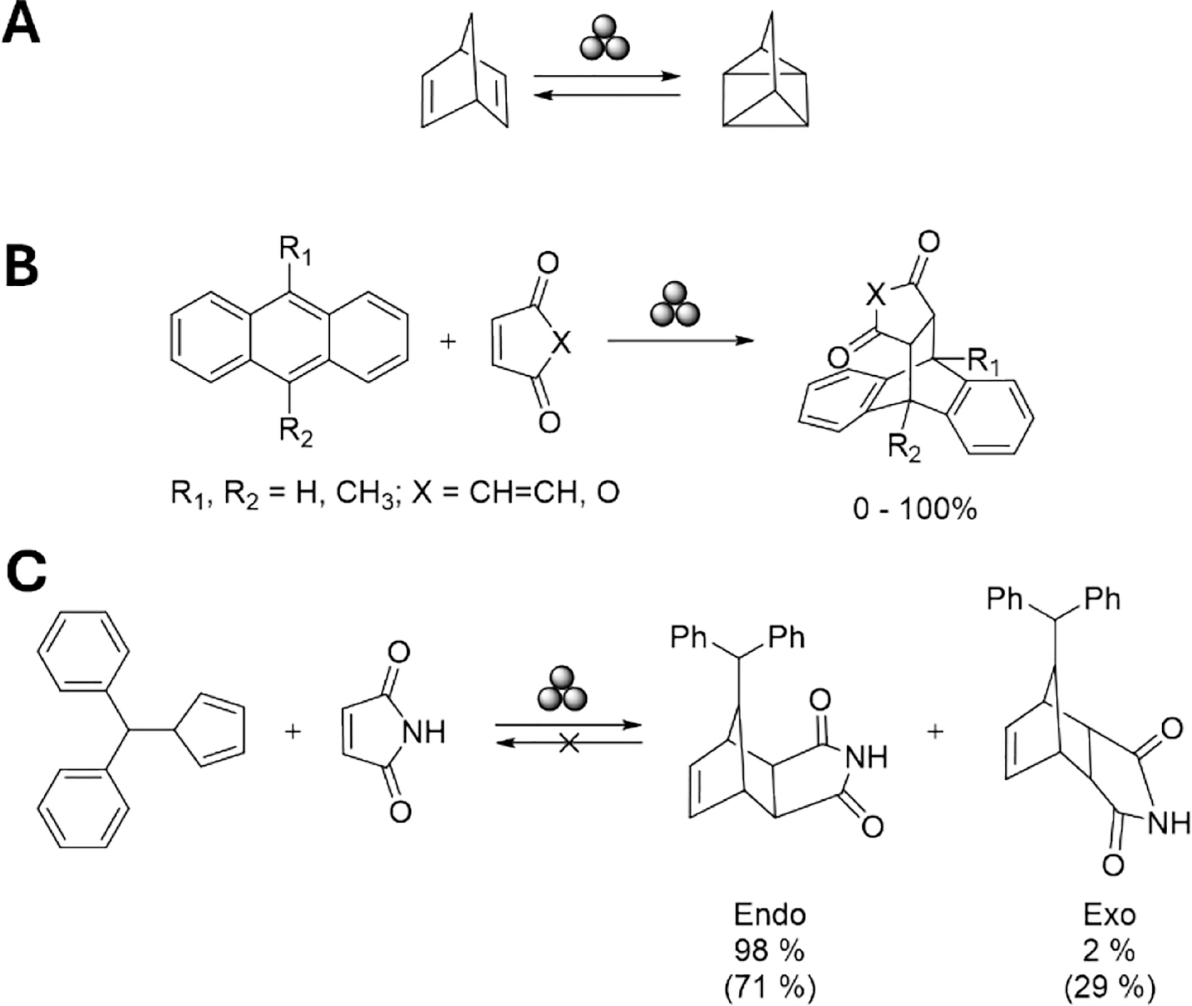
Reactions studied with the mechanochemical model. (A) Quadricyclane to norbornadiene reaction. (B) Different Diels–Alder reactions between anthracenes and maleic anhydride or benzoquinone. (C) Diphenylfulvene/maleimide Diels–Alder reaction. When available, the experimental ball-milling yields are given, while in parentheses, the thermally activated yields in solution are indicated.

#### NBD/QC [2 + 2] Cycloaddition

The [2 + 2] cycloaddition in the norbornadiene/quadricyclane (NBD/QC) system (Figure 4A) has been selected as a first case study due to its simplicity. This reaction is unimolecular, which makes it particularly suitable for testing the predictive capacity of the proposed method. In conventional applications, the NBD to QC conversion is commonly induced photochemically and has been studied in the context of molecular solar thermal (MOST) energy storage systems.^32^ Within this context, mechanically controlled reactions have also been explored, particularly covalent mechanochemistry under applied force.^33^ However, ball-milling-type forces have not been investigated in this system.

Here, we aim to evaluate how mechanical activation affects each reaction on the NBD ⇌ QC equilibrium. Specifically, we calculate the mechanical response for both the forward (NBD → QC) and reverse (QC → NBD) processes using our mechanochemical model.

The results obtained indicate that the NBD → QC process shows high mechanical susceptibility. The application of mechanical stress along the relevant compression directions leads to a significant decrease in the activation energy. This suggests that the cycloaddition step can be promoted under ball-milling conditions (see Figure 5). In fact, the activation energy can decrease up to more than 1.2 kcal mol^–1^ when 2 GPa impact pressures are considered (see Figure 5).

**Figure 5:**
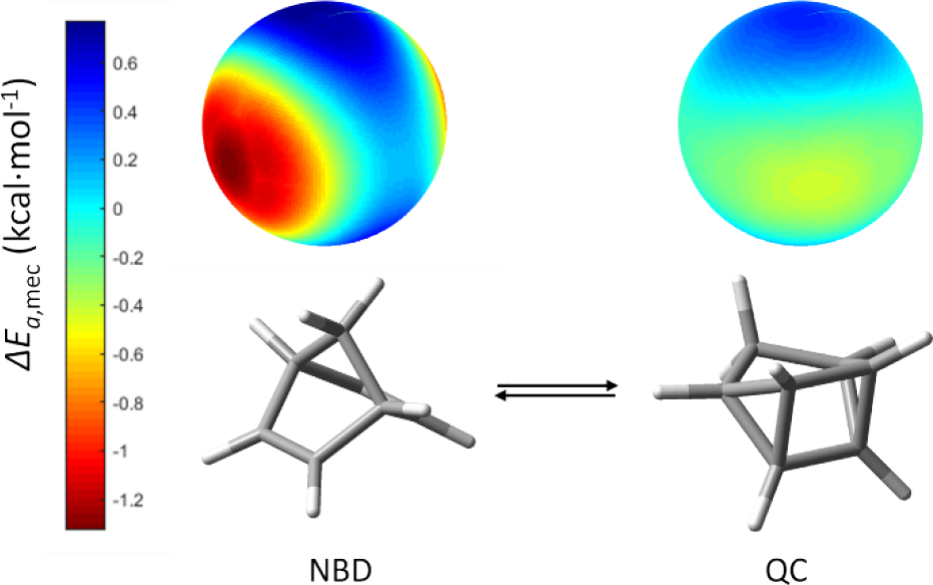
(left) Spherical representation of the activation energy difference in NBD, where activation energy decreases up to ca. 1.2 kcal mol^–1^ for 2 GPa average pressure impacts. This corresponds to a “hot” mechanochemical species. (right) Same representation for the QC → NBD reaction, where the sphere shows much lower response (“cold” mechanochemical species). The orientations of the two isomers are different for clarity.

In contrast, the QC → NBD back-reaction shows an almost negligible mechanical response (Figure 5). In this case, the activation barrier remains nearly unchanged upon the application of the same mechanical force, for which the forward process is strongly favored. This asymmetry in the mechanical response is the result of the nature of the ball-milling forces, which are compressing forces that selectively favor the cycloaddition instead of [2 + 2] cycloreversion.

These results show the intrinsic nature of ball-milling behavior that can affect a specific chemical reaction and have no effect on another one in contrast to conventional thermally activated processes.

#### Diels–Alder Reactions

To test the applicability of our model, we also selected a series of Diels–Alder reactions previously studied under ball-milling conditions by Andersen and Mack^34^ and later analyzed computationally by Pladevall et al.^26^ and by Sakai et al.^35^ These reactions involve combinations of anthracene derivatives and electron-deficient dienophiles, and depending on the set of reagents employed, variable yields from 0 to 100% were obtained experimentally under different experimental conditions (Figure 4B). The experimental trends have been successfully reproduced computationally using DFT-based microkinetic modeling,^26^ assuming that the mechanochemical acceleration arises from high local concentrations and modified dielectric environments. However, the effect of the mechanical force itself on the reaction coordinate was not explicitly considered. This makes these reactions a suitable benchmark for our model, which directly evaluates the mechanical contribution to the activation energy under isotropic compressive stress.

For the studied reactions, it is found that the mechanical compression leads to a modest but consistent decrease in the activation energy across the selected Diels–Alder systems. Specifically, the reaction between 9,10-dimethylanthracene (DMA) and maleic anhydride (MA) exhibits a Δ*E*_a_ of −0.48 kcal mol^–1^ under 1 GPa pressure. The reaction between 9-methylanthracene (MA9) and benzoquinone (BQ) shows almost the same decrease, with a Δ*E*_a_ of −0.51 kcal mol^–1^, as well as the reaction between anthracene (H9) and BQ with a Δ*E*_a_ of −0.50 kcal mol^–1^ (see Figure 6).

**Figure 6:**
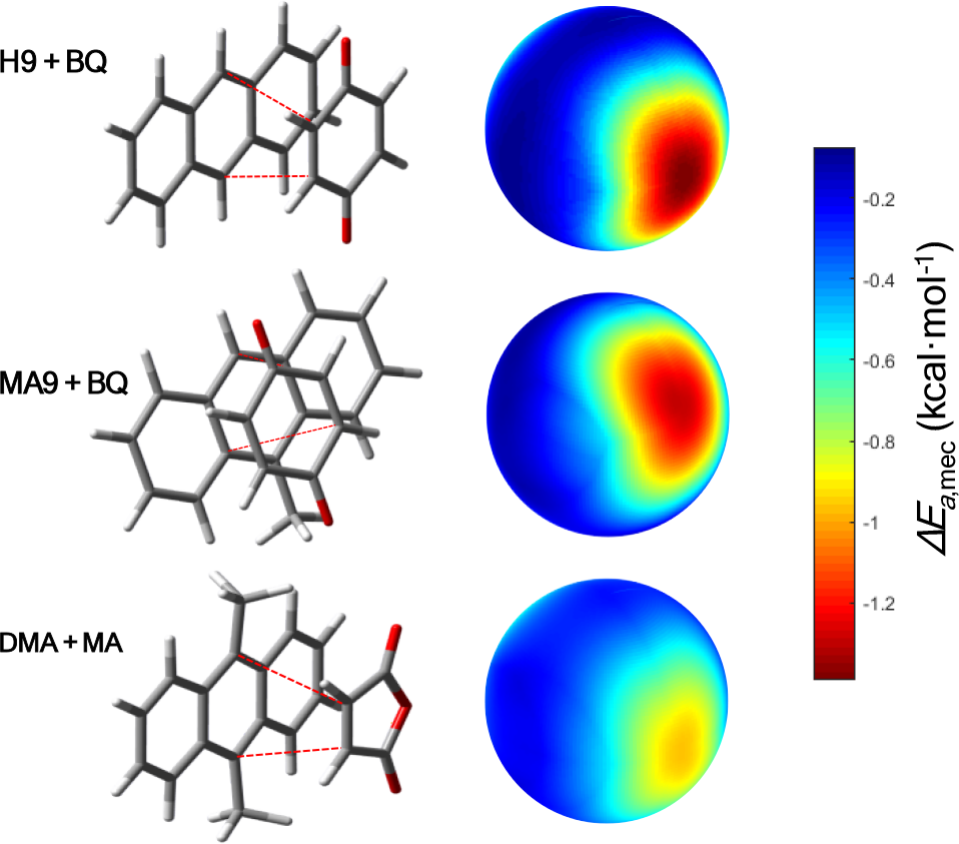
(left) Optimized geometries of the three Diels–Alder adducts for the reactions studied: H9 + BQ, MA9 + BQ, and DMA + MA. (right) Spatial distribution of mechanical variation of the activation energy (same orientation as the corresponding molecular structure) for a 1 GPa impact pressure.

The mechanical effect is quite similar across all three reactions, producing a nearly uniform decrease in the activation energy. Consequently, since the activation energies in the absence of mechanical stress are 25.8 kcal mol^–1^ for H9 + BQ, 22.8 kcal mol^–1^ for MA9 + BQ, and 17.9 kcal mol^–1^ for DMA + MA,^26^ the order of reactivity remains essentially unchanged under ball milling.^34^ That is, the mechanical input enhances the reaction rates uniformly, and the system with the lowest intrinsic barrier (DMA + MA) continues to be the most favored, followed by MA9 + BQ and then H9 + BQ.

#### Endo- vs Exoselectivity in a Diphenylfulvene/Maleimide Diels–Alder Reaction

As a final case study, we have investigated the Diels–Alder reaction between diphenylfulvene and maleimide (Figure 4C) since the kinetics of this transformation has been experimentally^36^ studied both under mechanochemical conditions using a ball mill and in toluene solution. Shorter reaction times and lower temperatures were needed in the mechanochemical experiments compared to the conventional solution-based reactivity. In addition, independently of the experimental conditions used in the ball milling experiments, the reaction between diphenylfulvene and maleimide always leads to a constant selectivity between the two possible cycloaddition *endo* and *exo* products (98/2 ratio, see Figure 4C) even at different temperatures. However, the *endo*/*exo* ratio obtained in toluene solution decreases with longer reaction times and at higher temperatures, indicating that the *endo* is the kinetically favored compound, while the *exo* is the thermodynamically preferred species, and a slow chemical equilibrium between both occurs through the retro-Diels–Alder reaction. Moreover, this reactivity has been previously studied also computationally.^27^

Using the ball-milling model developed in this work, we evaluated the mechanical activation of the two possible cycloaddition pathways leading to the *endo* and *exo* products. Wall-type compressive forces corresponding to a local pressure of approximately 1.0 GPa were applied to the reactant complex, corresponding to approximately 1.4 nN applied to the reactive complex. The mechanical work performed along the reaction coordinate for both activation processes (*endo* and *exo* reaction pathways) was calculated. The results indicate that mechanical activation significantly favors the formation of the *endo* adduct. The activation energy variation due to the mechanical effect, ⟨Δ*E*_a,mec_⟩, for the *endo* pathway is ca. −3.88 kcal mol^–1^. In contrast, this energy variation is −2.90 kcal mol^–1^ for the *exo* pathway. This behavior is consistent with the experimental findings reported by Gonnet et al., where the *endo* product is the major species formed under ball-milling conditions.^36^ The mechanochemical model captures this selective reactivity, demonstrating that the application of isotropic compressive forces can selectively enhance the formation of the *endo* product. A spherical representation of the activation energy variation under compression forces for both the *endo* and *exo* pathways is shown in Figure 7.

**Figure 7:**
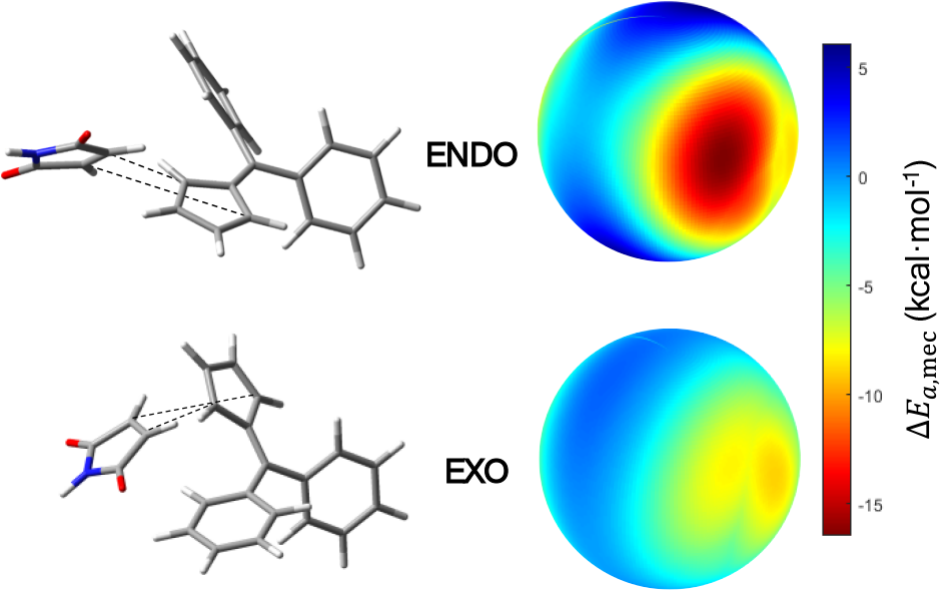
(up) *Endo* adduct and the corresponding spherical representation of Δ*E*_a,mec_ as a function of the direction of wall-type forces. Some impact directions permit activation energy variations of ca. −16.5 kcal mol^–1^, being the average ⟨Δ*E*_a,mec_⟩ = −3.88 kcal mol^–1^. (down) Same representation for the *exo* case. The lower activation energy variation is ca. −8.9 kcal mol^–1^, being the ⟨Δ*E*_a,mec_⟩ = −2.89 kcal mol^–1^.

The map highlights that the mechanical work for the *endo* pathway is significant, therefore enhancing this reaction pathway, while the *exo* pathway exhibits a much lower mechanical response. Overall, this case study confirms that mechanical forces can selectively favor specific reaction pathways using ball milling and supports the predictive capacity of the proposed model.

Furthermore, the retro-Diels–Alder reaction was analyzed by using our mechanochemical model for ball milling. For the *endo* Diels–Alder reaction, the model predicts a significant mechanical effect. The activation energy is lowered by ∼4 kcal mol^–1^ under 1 GPa of effective pressure. On the contrary, the mechanical contribution to the retro-Diels–Alder pathway is almost negligible since the activation barrier remains nearly unaffected under mechanical stress (see Figure 8). These results are again consistent with experimental findings reported by Gonnet et al.,^36^ where no equilibrium is observed between the *endo* and the *exo* species under ball-milling conditions since the reverse reaction that would allow their interconversion is not activated mechanochemically as predicted by our model.^36^

**Figure 8:**
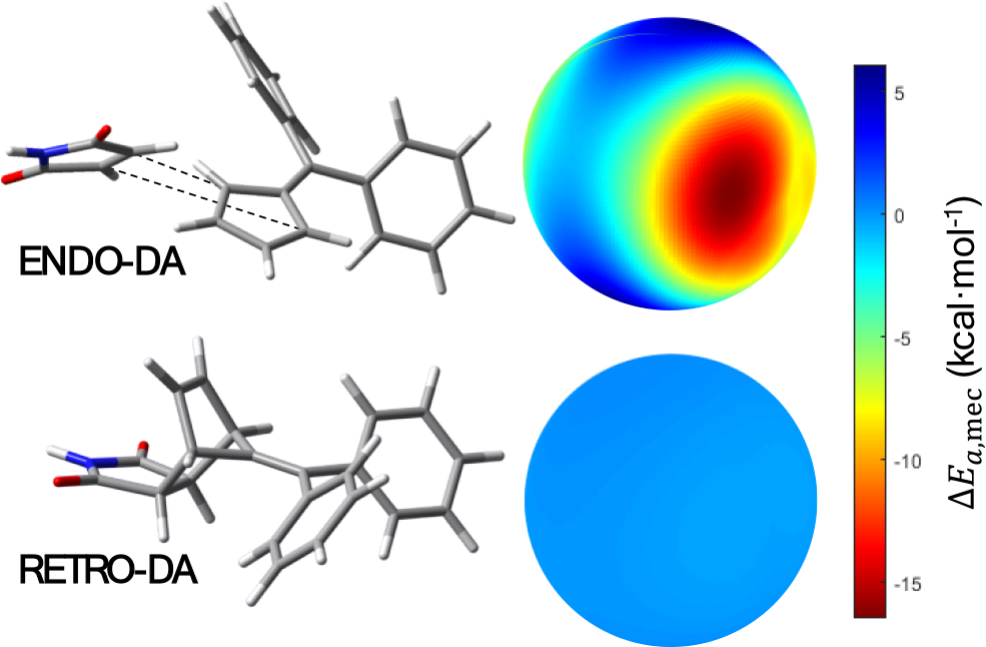
Comparison between the activation energy variation for the *endo*-DA reaction (up) and retro-Diels–Alder reaction from the *endo* product (down). The variation of the activation energy due to the mechanical effect in the retro-DA reaction is almost negligible, as is apparent from the scale representation of Δ*E*_a,mec_.

It is worth noting that the Diels–Alder reaction between diphenylfulvene and maleimide have a larger mechanical effect than equivalent Diels–Alder reactions involving H9, MA9, or DMA. Although the mechanical effect in all of them favors the reaction, the first case involves an adduct structure, which is farthest to the transition state than in the rest of the cases. This situation implies larger changes in nuclear coordinates from the minimum to the transition state, and therefore, the applied forces develop larger mechanical work along the reaction path.

## Conclusions

We developed a mechanochemical model and the corresponding computational algorithm to predict the mechanical activation of chemical reactions under ball-milling conditions. The model simplifies the complex forces in ball-milling by considering isotropic compression, mimicking ball collisions, and quantifying the work performed along the reaction path. The model computes the mechanically induced variation in the activation energy, identifying whether a reaction can be promoted by ball milling. The predictions of the model can be, of course, straightforward complemented with additional refinements, as considering the dielectric constant of the solid reactants as has been proposed by Maseras group.^26^ Their approach determines both the change in activation energy due to the altered dielectric environment and the corresponding optimized geometries of the reactants and transition state in that medium. Because the mechanical work correction in our model depends only on these geometries, it can be readily evaluated by using the same structures and then summed directly to the dielectric correction.

Our results have been tested in different case studies, demonstrating the model’s reliability in predicting mechanochemical activation. The simple and intuitive norbornadiene to quadricyclane transformation illustrates a strong differential mechanical response (the cyclization reaction is significantly favored regarding the ring opening process), highlighting the selectivity of mechanical activation. This behavior is also found in the Diels–Alder reaction between diphenylfulvene and maleimide, where the high selectivity in the formation of the *endo* product found in ball-milling reactivity is also predicted by our model. Additionally, the model also accounts for the mechanical effect boosting the formation of the *endo* product and avoiding the retro-Diels–Alder reaction.

The proposed model is simple to implement (it only needs the adduct and transition state structures as input) and effectively captures the influence of directional compression on the chemical reactivity. It offers a straightforward and efficient tool for reaction screening, enabling the identification of transformations that may benefit from ball milling, a technique increasingly employed in research laboratories as a sustainable and accelerated alternative to conventional synthetic methods.

Furthermore, due to its simple implementation and low computational cost, it could be used to explore new applications of ball-milling in areas like green chemistry and material synthesis, where work is currently in progress in our group.

## Supplementary Material



## Notes

Study of grid density in numerical integration and geometries of minima and transition states (PDF). Python code for the developed Bell-type ball milling mechanochemical model is available at: https://github.com/LMFrutos/Bell_BallMilling. Input/Output files of the systems studied with the Bell Ball-Milling model software are available at: https://doi.org/10.5281/zenodo.15792874
