# Supplementary material for: Computational Model to Predict Reactivity under Ball-Milling Conditions
Source: J Chem Theory Comput. 2025 Jul 17;21(20):10353–61. doi: 10.1021/acs.jctc.5c00832 (PMC12573735; doi:10.1021/acs.jctc.5c00832)
Supplement: Supplementary file 1 [file ct5c00832_si_001.pdf]

Supporting Information

A Computational Model to Predict Reactivity under Ball-Milling Conditions

Raúl De Armas,[a] Manuel Temprado[a,b,] and Luis Manuel Frutos[a,b,*]*

[a] Universidad de Alcalá, Departamento de Química Analítica, Química Física e Ingeniería Química, Grupo de Reactividad y Estructura Molecular (RESMOL), Alcalá de Henares, Madrid, Spain.
[b] Universidad de Alcalá, Instituto de Investigación Química "Andrés M. del Río" (IQAR), Alcalá de Henares, Madrid, Spain.

Content

1. Analysis of $\langle\Delta E_a\rangle$ with angular grid (θ and ϕ)……………………………………………………..S1

2. Cartesian Coordinates of different molecular geometries…………………………………………..S4

3. References………………………………………………………………………………………S16

1. Analysis of $\langle\Delta E_a\rangle$ with angular grid (θ and ϕ)

The predictions of the algorithm developed to determine ΔE_a depends on the grid selected. Nevertheless, the procedure is quite robust, and since the variation of the activation energy is smooth. We have chosen for our calculations $n_\theta = 100$, and $n_\phi = 2\,n_\theta = 200$. The choice $n_\phi = 2\,n_\theta$ forces the relation $\Delta\phi = \Delta\theta$, i.e. same variation of the angle θ and ϕ in the grid.

In order to show the convergence behavior, we chose as an example the NBD/QC case. For this system, a series of numerical integrations has been performed, going from $n_\theta = 10$ up to $n_\theta = 500$ (see Table S1). The stability of the numerical integration is reached very fast. Even the lowest resolved grid ($n_\theta = 10$ and $n_\phi = 20$), shows a relatively small error of ca. 2%. This numerical integration is computationally inexpensive, and performing fine grid integrations (e.g. N=500, which means $5{\cdot}10^5$ steps of integration) remains within just few seconds in a standard personal computer. Table S1 shows the results of the series of numerical integrations. Figure S1 displays the $\langle\Delta E_a\rangle$ calculated as a function of $\boldsymbol{log}(\,n_\theta\,n_\phi) = log(2N^2)$. Finally, Figure S2 displays the determined maps of activation energy as a function of θ and ϕ.

$n_\theta \equiv N$	n_ϕ	$n_\theta \cdot n_\phi$	$log(n_\theta\, n_\phi)$	$\langle\Delta E_a\rangle$ $(kcal/mol)$
**500**	1000	500000	5.699	-0.1972
**300**	600	180000	5.255	-0.1972
**200**	400	80000	4.903	-0.1972
**100**	200	20000	4.301	-0.1972
**80**	160	12800	4.107	-0.1973
**60**	120	7200	3.857	-0.1973
**50**	100	5000	3.699	-0.1973
**40**	80	3200	3.505	-0.1974
**30**	60	1800	3.255	-0.1977
**20**	40	800	2.903	-0.1981
**10**	20	200	2.301	-0.2009

Table S1. Variation of the average activation energy $\langle\Delta E_a\rangle$ with the integration grid. The number of steps of integrations in θ is the control parameter: $n_\theta \equiv \boldsymbol{N}$. The number of steps of integrations in ϕ (i.e. n_ϕ) always fulfil $n_\phi = 2\, n_\theta$. As can be seen, the variation of $\langle\Delta E_a\rangle$ rapidly converged with *N*. The smaller grid (with *N*=10 and just 200 points of integration) has an error in $\langle\Delta E_a\rangle$ of ca. 2%. The error is reduced to 0.5% in the case of *N*=20, and >0.001% error in case of *N*=100 (used in the manuscript).

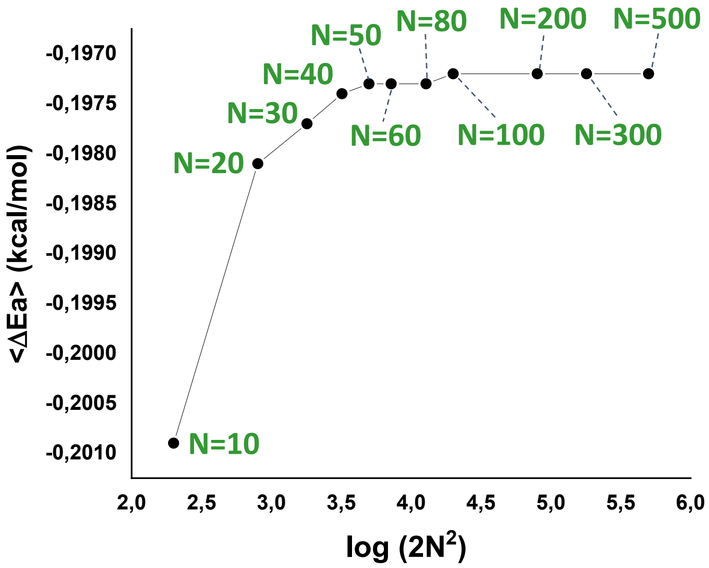

Fig S1. Variation of $\langle\Delta E_a\rangle$ with the integration grid (logarithmic scale) given by $\boldsymbol{log}(n_\theta \cdot n_\phi) = log(2N^2)$. A rapid convergence is reached for small grid of integration.

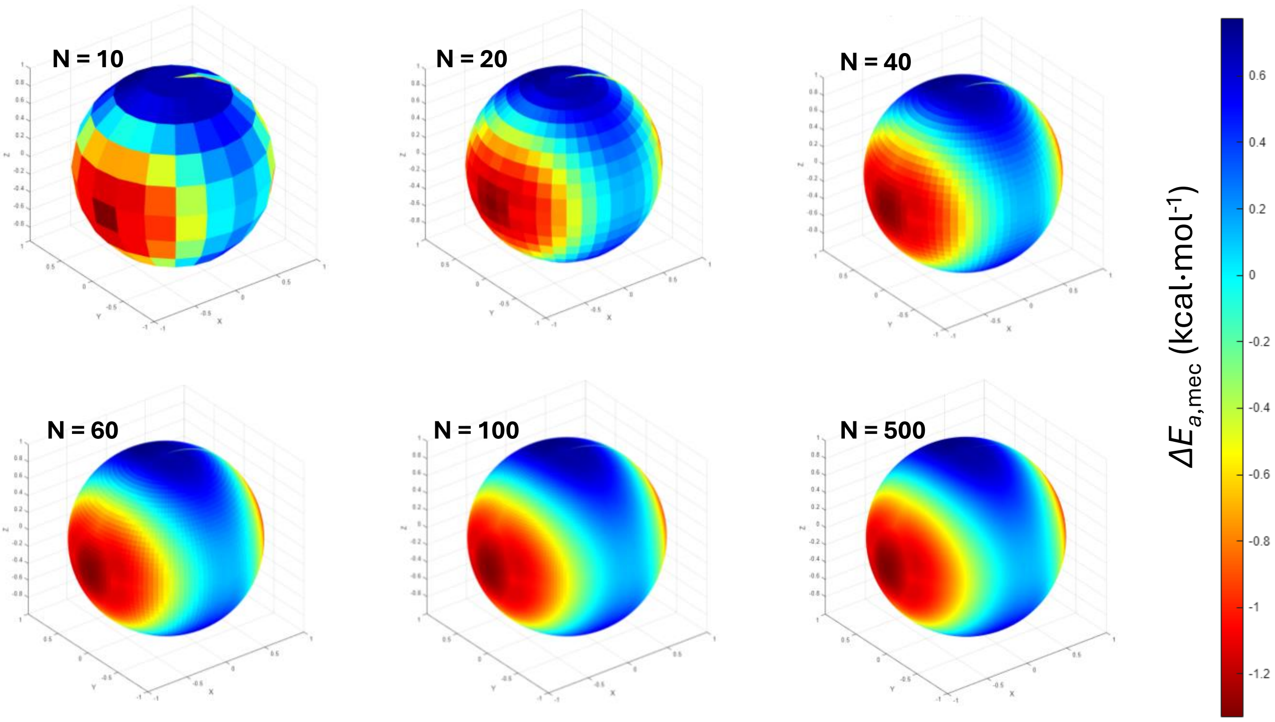

Fig S2. Variation of the integration grid in the case of NBD/QC. Despite the low resolution of the low resolved case (N=10), the error in $\langle \Delta E_a \rangle$ is only ca. 2%.

2. Cartesian Coordinates of different molecular geometries.

NBD (geometry taken from reference S1 -page S8 of Supporting Info.-):

```
C 0.000010 0.000031 1.348548
C 0.000199 1.117913 0.276665
C -0.000199 -1.117900 0.276711
C 1.237929 -0.671425 -0.519215
C 1.238271 0.670999 -0.519081
C -1.237942 0.671400 -0.519222
C -1.238269 -0.671022 -0.519057
H -0.888705 0.000202 1.969265
H 0.888738 -0.000110 1.969249
H 0.000350 2.143119 0.621243
H -0.000349 -2.143092 0.621330
H 1.910863 -1.330929 -1.033435
H 1.911536 1.330268 -1.033170
H -1.910893 1.330881 -1.033449
H -1.911532 -1.330311 -1.033121
```

TS NBD/QC (geometry taken from reference S1 -page S8 of Supporting Information-):

```
C 0.459205 1.369593 0.000027
C 1.203822 0.018540 -0.000362
C -0.950234 0.782813 0.000372
C -0.886735 -0.438343 0.863542
C 0.524124 -0.685436 1.171992
C 0.523446 -0.685291 -1.172475
C -0.887240 -0.437989 -0.863266
H 0.665385 1.960415 -0.886403
H 0.665932 1.960189 0.886477
H 2.283275 0.052145 -0.000742
H -1.821589 1.413913 0.000789
H -1.728888 -0.836623 1.394598
H 0.874300 -1.628487 1.555323
H 0.872941 -1.629003 -1.554866
H -1.729689 -0.835877 -1.394152
```

QC (geometry taken from reference S1 -page S8 of Supporting Information-):

```
C -0.000001 1.529340 -0.000015
C -1.144866 0.538712 0.000014
C 1.144865 0.538714 -0.000024
C 0.770915 -0.702097 -0.766013
C -0.770914 -0.702062 -0.766031
C -0.770914 -0.702083 0.766027
C 0.770915 -0.702047 0.766045
H 0.000016 2.161792 0.883140
H -0.000019 2.161775 -0.883183
H -2.170581 0.863753 0.000026
H 2.170579 0.863756 -0.000042
H 1.413523 -1.210536 -1.460107
H -1.413528 -1.210459 -1.460151
H -1.413521 -1.210511 1.460130
H 1.413530 -1.210430 1.460174
```

DMA+MA (min) (geometry taken from reference S2 -page S23 of Supporting Information. Label: add-dma9-ma-gp-):

C -2.7884 0.7080 2.0967
H -3.6708 1.2468 2.4219
C -2.7883 -0.7087 2.0966
H -3.6707 -1.2477 2.4217
C -1.6835 -1.3967 1.6766
H -1.7105 -2.4769 1.6745
C -0.5035 -0.7207 1.2340
C -0.5035 0.7203 1.2342
C -1.6836 1.3961 1.6768
H -1.7108 2.4763 1.6749
C 0.6291 -1.4368 0.7806
C 1.7996 -0.7248 0.4317
C 1.7994 0.7247 0.4316
C 0.6288 1.4366 0.7806
C 3.0089 -1.3929 0.0532
H 3.0440 -2.4728 0.0638
C 4.1375 -0.7087 -0.3036
H 5.0369 -1.2495 -0.5755
C 4.1373 0.7090 -0.3039
H 5.0365 1.2499 -0.5761
C 3.0085 1.3930 0.0527
H 3.0433 2.4729 0.0628
C -0.1604 0.6677 -2.1453
H 0.6431 1.3540 -2.3600
C -0.1620 -0.6688 -2.1451
H 0.6398 -1.3571 -2.3597
C -1.5187 -1.1295 -1.7642
C -1.5159 1.1317 -1.7644
O -2.3040 0.0021 -1.5522
O -1.9486 -2.2373 -1.6370
O -1.9432 2.2405 -1.6372
C 0.5474 2.9436 0.7027
H 0.4616 3.3871 1.7001
H -0.3328 3.2469 0.1293
H 1.4103 3.3937 0.2197
C 0.5477 -2.9438 0.7022
H -0.3327 -3.2469 0.1289
H 0.4621 -3.3878 1.6994
H 1.4105 -3.3936 0.2189

DMA+MA (TS) (geometry taken from reference S2 -page S20 of Supporting Information. Label: ts-dma9-ma-gp):

C -2.6157 0.7019 2.3697
H -3.4074 1.2429 2.8750
C -2.6157 -0.7019 2.3697
H -3.4074 -1.2429 2.8750
C -1.6160 -1.3971 1.7185
H -1.6374 -2.4786 1.7130
C -0.5643 -0.7107 1.0764
C -0.5643 0.7107 1.0764
C -1.6160 1.3971 1.7185
H -1.6374 2.4786 1.7130
C 0.4775 -1.3881 0.3266
C 1.7632 -0.7097 0.2869
C 1.7632 0.7097 0.2869
C 0.4775 1.3881 0.3266
C 2.9902 -1.3952 0.1801
H 3.0062 -2.4771 0.1847
C 4.1841 -0.7016 0.1038
H 5.1213 -1.2436 0.0500
C 4.1841 0.7016 0.1038
H 5.1213 1.2436 0.0500
C 2.9902 1.3952 0.1801
H 3.0062 2.4771 0.1847
C -0.1121 0.7064 -1.6752
H 0.6300 1.3285 -2.1510
C -0.1121 -0.7064 -1.6752
H 0.6300 -1.3285 -2.1510
C -1.5250 -1.1376 -1.6855
C -1.5250 1.1376 -1.6855
O -2.3247 0.0000 -1.5600
O -2.0055 -2.2322 -1.7594
O -2.0055 2.2322 -1.7594
C 0.4343 2.8908 0.2038
H 0.7127 3.3594 1.1543
H -0.5610 3.2351 -0.0734
H 1.1238 3.2458 -0.5622
C 0.4343 -2.8908 0.2038
H -0.5610 -3.2351 -0.0734
H 0.7127 -3.3594 1.1543
H 1.1238 -3.2458 -0.5622

H9+BQ (min) (geometry taken from reference S2 -page S33 of Supporting Information. Label: add-h9-bq-gp):

C 2.6969 2.2140 -0.7125
H 3.5958 2.5033 -1.2452
C 2.6966 2.2144 0.7114
H 3.5953 2.5040 1.2444
C 1.5771 1.8446 1.4054
H 1.5799 1.8296 2.4899
C 0.3832 1.4622 0.7200
C 0.3837 1.4615 -0.7217
C 1.5778 1.8436 -1.4068
H 1.5811 1.8279 -2.4913
C -0.7729 1.0681 -1.4035
H -0.7641 1.0472 -2.4886
C -1.9324 0.6832 -0.7221
C -1.9329 0.6841 0.7198
C -0.7738 1.0697 1.4015
H -0.7657 1.0502 2.4867
C -3.1123 0.2600 1.4049
H -3.1104 0.2593 2.4899
C -4.2199 -0.1439 0.7111
H -5.1081 -0.4659 1.2430
C -4.2194 -0.1449 -0.7140
H -5.1071 -0.4676 -1.2461
C -3.1113 0.2580 -1.4075
H -3.1085 0.2559 -2.4925
C 0.0345 -2.0367 -0.6656
H -0.8472 -2.2786 -1.2470
C 0.0371 -2.0351 0.6743
H -0.8423 -2.2757 1.2597
C 1.2489 -1.6825 -1.4401
C 2.4681 -1.3282 -0.6706
H 3.3385 -1.0636 -1.2590
C 2.4706 -1.3265 0.6685
H 3.3432 -1.0603 1.2530
C 1.2542 -1.6786 1.4434
O 1.2476 -1.6804 -2.6616
O 1.2572 -1.6729 2.6649

H9+BQ (TS) (geometry taken from reference S2 -page S21 of Supporting Information. Label: ts-h9-bq-gp):

C 2.5354 2.4161 -0.7098
H 3.3412 2.9019 -1.2480
C 2.5354 2.4195 0.6980
H 3.3412 2.9080 1.2338
C 1.5216 1.7947 1.3992
H 1.5310 1.7730 2.4833
C 0.4560 1.1864 0.7057
C 0.4560 1.1829 -0.7115
C 1.5215 1.7879 -1.4079
H 1.5309 1.7609 -2.4919
C -0.5944 0.4315 -1.3493
H -0.5569 0.3243 -2.4289
C -1.8861 0.3964 -0.7093
C -1.8861 0.3998 0.7074
C -0.5944 0.4381 1.3472
H -0.5568 0.3360 2.4273
C -3.0995 0.2424 1.4027
H -3.0944 0.2271 2.4873
C -4.2870 0.1225 0.7037
H -5.2235 0.0251 1.2408
C -4.2870 0.1191 -0.7041
H -5.2235 0.0191 -1.2407
C -3.0995 0.2356 -1.4037
H -3.0945 0.2150 -2.4882
C 0.0001 -1.5529 -0.7083
H -0.8234 -2.0238 -1.2303
C 0.0001 -1.5494 0.7158
H -0.8234 -2.0178 1.2402
C 1.2815 -1.5946 -1.4498
C 2.5195 -1.3506 -0.6669
H 3.4237 -1.2090 -1.2482
C 2.5195 -1.3474 0.6733
H 3.4238 -1.2029 1.2539
C 1.2816 -1.5875 1.4575
O 1.3350 -1.7998 -2.6544
O 1.3351 -1.7868 2.6631

MA9+BQ (min) (geometry taken from reference S2 -page S23 of Supporting Information. Label: add-ma9-bq-gp):

C 2.6989 0.0857 2.3629
H 3.6044 0.4776 2.8119
C 2.6434 -1.2751 1.9583
H 3.5108 -1.9102 2.0999
C 1.5115 -1.7900 1.3846
H 1.5090 -2.8244 1.0694
C 0.3450 -0.9853 1.1790
C 0.4136 0.3957 1.5846
C 1.6148 0.8965 2.1734
H 1.6493 1.9417 2.4611
C -0.6890 1.2264 1.3771
H -0.6288 2.2705 1.6663
C -1.8597 0.7456 0.7903
C -1.9419 -0.6386 0.3819
C -0.8358 -1.4978 0.5872
C -3.1578 -1.0583 -0.2493
H -3.2645 -2.0817 -0.5805
C -4.1955 -0.1917 -0.4615
H -5.0991 -0.5436 -0.9468
C -4.1048 1.1673 -0.0524
H -4.9375 1.8391 -0.2267
C -2.9685 1.6163 0.5582
H -2.8828 2.6509 0.8735
C 0.1804 1.4072 -1.7474
H -0.6621 2.0837 -1.8270
C 0.1093 0.1197 -2.1151
H -0.7958 -0.3237 -2.5124
C 1.4273 1.9820 -1.1883
C 2.5916 1.0721 -1.0457
H 3.4867 1.5117 -0.6221
C 2.5196 -0.2140 -1.4113
H 3.3506 -0.9012 -1.3067
C 1.2716 -0.7870 -1.9735
O 1.4976 3.1564 -0.8587
O 1.2082 -1.9631 -2.3040
C -0.8671 -2.9496 0.1741
H -0.1869 -3.1250 -0.6663
H -1.8556 -3.2834 -0.1291
H -0.5521 -3.5958 0.9976

MA9+BQ (TS) (geometry taken from reference S2 -page S25 of Supporting Information. Label: ts-ma9-bq-gp):

C 2.5286 0.0762 2.5787
H 3.3391 0.4441 3.1972
C 2.4517 -1.2921 2.2647
H 3.2030 -1.9743 2.6462
C 1.4283 -1.7754 1.4713
H 1.3904 -2.8304 1.2330
C 0.4210 -0.9070 0.9946
C 0.5070 0.4721 1.3126
C 1.5775 0.9515 2.0903
H 1.6415 2.0127 2.3038
C -0.4672 1.3415 0.6955
H -0.3770 2.4057 0.8914
C -1.7999 0.8080 0.5296
C -1.8980 -0.5705 0.2172
C -0.6628 -1.3304 0.1302
C -3.1728 -1.1128 -0.0504
H -3.2772 -2.1646 -0.2832
C -4.3020 -0.3156 0.0085
H -5.2775 -0.7511 -0.1754
C -4.1940 1.0542 0.3038
H -5.0844 1.6712 0.3416
C -2.9518 1.6121 0.5467
H -2.8565 2.6704 0.7655
C 0.1126 1.2625 -1.2655
H -0.6605 1.9420 -1.6056
C 0.0563 -0.0900 -1.7121
H -0.8033 -0.4332 -2.2725
C 1.4390 1.9272 -1.1086
C 2.6308 1.0475 -1.0841
H 3.5596 1.5145 -0.7761
C 2.5571 -0.2371 -1.4580
H 3.4254 -0.8865 -1.4766
C 1.2897 -0.8432 -1.9468
O 1.5425 3.1363 -0.9685
O 1.3115 -1.9303 -2.5186
C -0.7086 -2.7522 -0.3544
H 0.2673 -3.0904 -0.6995
H -1.3973 -2.8653 -1.1911
H -1.0376 -3.4160 0.4543

ENDO-FORWARD (MIN) (geom. optimized B3LYP/6-311G(d,p)):

6	-5.518540	-0.991659	-1.354798
6	-5.054272	-1.524931	-0.152788
6	-3.828336	-1.121719	0.364103
6	-3.051258	-0.156027	-0.296601
6	-3.527217	0.365925	-1.509368
6	-4.748220	-0.050464	-2.033484
6	-1.748173	0.278628	0.272720
6	-0.831335	-0.801267	0.719888
6	-1.419365	1.601659	0.373406
6	-0.134140	-0.717688	1.935706
6	0.717145	-1.741708	2.341771
6	0.887898	-2.871668	1.545247
6	0.194793	-2.974755	0.338700
6	-0.662219	-1.956267	-0.064699
6	-0.108649	2.176750	0.722048
6	3.169919	-0.512279	-0.669354
6	4.074886	-0.900949	-1.568461
6	-2.316626	2.748758	0.152470
6	-1.606968	3.883738	0.360119
6	-0.225340	3.526589	0.696705
6	5.367245	-0.177432	-1.312281
6	3.800527	0.499183	0.247704
7	5.112368	0.636866	-0.204970
8	6.408171	-0.262588	-1.912518
8	3.302820	1.081649	1.179918
1	-5.647996	-2.258052	0.381675
1	-3.471836	-1.543151	1.296770
1	-2.924131	1.087842	-2.046035
1	-6.470379	-1.313420	-1.762079
1	-5.094601	0.358066	-2.976257
1	-0.276011	0.148930	2.568334
1	1.548117	-3.670085	1.864755
1	1.243535	-1.656003	3.285364
1	0.316913	-3.853246	-0.285276
1	-1.203797	-2.046945	-0.998927
1	5.790584	1.258771	0.207739
1	2.144230	-0.829282	-0.549012
1	3.974283	-1.611807	-2.375476
1	-1.989020	4.894546	0.305965
1	0.569699	4.236133	0.883241
1	-3.370139	2.674391	-0.066690
1	0.795518	1.618272	0.908974

ENDO-FORWARD (TS) (geom. optimized B3LYP/6-311G(d,p)):

6	-3.254328	3.696287	-0.177623
6	-3.778811	2.637603	0.562409
6	-3.069592	1.447722	0.682364
6	-1.808445	1.296645	0.083828
6	-1.298970	2.366684	-0.665185
6	-2.015089	3.553730	-0.796520
6	-1.066106	0.014002	0.241760
6	-1.851492	-1.231728	0.011635
6	0.236775	-0.036218	0.611512
6	-1.871468	-2.275344	0.948390
6	-2.609962	-3.432344	0.710695
6	-3.340108	-3.567980	-0.467143
6	-3.340009	-2.532425	-1.401062
6	-2.614444	-1.371361	-1.158801
6	1.104962	-1.226060	0.773026
6	2.189170	-0.619185	-0.980222
6	2.249006	0.784450	-0.827511
6	1.134196	1.059296	1.037307
6	2.053459	0.482142	1.944820
6	2.036616	-0.897481	1.787033
6	3.647203	1.153022	-0.466599
6	3.554690	-1.167172	-0.731416
7	4.320076	-0.067497	-0.319256
8	4.141076	2.246973	-0.321997
8	3.959862	-2.300489	-0.840038
1	-4.744564	2.737828	1.044879
1	-3.487899	0.627045	1.253229
1	-0.346884	2.261845	-1.168924
1	-3.810503	4.621422	-0.276812
1	-1.604927	4.364686	-1.387716
1	-1.323426	-2.165142	1.876372
1	-3.912836	-4.469389	-0.653000
1	-2.619115	-4.224560	1.450841
1	-3.909434	-2.628411	-2.318673
1	-2.627314	-0.566270	-1.884463
1	5.301368	-0.132730	-0.091022
1	1.536075	-1.142669	-1.662739
1	1.658877	1.498550	-1.380213
1	2.741968	1.041714	2.563040
1	2.714576	-1.597547	2.255327
1	0.855473	2.102605	1.046910
1	0.807023	-2.234091	0.527843

EXO-REVERSE (min) (geom. optimized B3LYP/6-311G(d,p)):

6	-2.103821	4.114686	-0.163808
6	-3.204438	3.273310	-0.000541
6	-3.017730	1.910235	0.195450
1	-4.209197	3.679804	-0.030166
6	-1.724070	1.364786	0.262745
1	-3.876538	1.260269	0.315801
6	-0.624291	2.220365	0.088831
6	-0.817309	3.582564	-0.126832
1	0.381514	1.819340	0.088064
1	-2.250605	5.176320	-0.328571
1	0.044805	4.223031	-0.274216
6	-1.540994	-0.090347	0.490049
6	-2.391959	-1.001196	-0.316072
6	-0.638576	-0.570391	1.401357
6	-3.064907	-2.084454	0.271580
6	-3.861742	-2.928340	-0.497020
1	-2.976764	-2.244931	1.338850
6	-3.997033	-2.710562	-1.866205
1	-4.615984	-3.369738	-2.464364
1	-4.383027	-3.752655	-0.023349
6	-3.342016	-1.632996	-2.461694
6	-2.559760	-0.778793	-1.693246
6	-0.210942	-1.966309	1.577996
6	4.219750	-1.383097	-0.222511
6	3.174974	-0.589469	0.018635
6	0.088919	0.203556	2.416990
1	-3.444713	-1.455683	-3.526355
1	-2.058509	0.060886	-2.159924
6	0.854875	-0.655971	3.135895
6	0.682797	-2.007512	2.599934
6	3.451305	0.764694	-0.573691
6	5.259338	-0.614661	-0.987625
7	4.715253	0.663450	-1.152327
8	2.749278	1.747405	-0.568370
8	6.333119	-0.989031	-1.385146
1	5.178887	1.417565	-1.635314
1	4.367594	-2.416405	0.054975
1	2.259563	-0.811318	0.548543
1	1.481876	-0.400071	3.979490
1	1.195186	-2.888033	2.964783
1	-0.032430	1.261475	2.588268
1	-0.521828	-2.791600	0.956582

EXO-REVERSE (TS) (geom. optimized B3LYP/6-311G(d,p)):

6	-2.847055	3.661046	-0.104281
6	-3.427825	2.547668	0.500509
6	-2.693036	1.379062	0.664226
1	-4.456354	2.587092	0.841380
6	-1.352191	1.302949	0.249808
1	-3.155282	0.516250	1.128799
6	-0.782454	2.428622	-0.365144
6	-1.526495	3.593032	-0.540704
1	0.233793	2.400224	-0.733991
1	-3.421898	4.569975	-0.241545
1	-1.068508	4.445680	-1.029145
6	-0.602348	0.033716	0.472953
6	-1.339380	-1.219599	0.142770
6	0.640267	-0.007834	1.021724
6	-1.507487	-2.245630	1.083242
6	-2.207577	-3.404414	0.754851
1	-1.107956	-2.120328	2.082438
6	-2.746767	-3.559101	-0.519180
1	-3.286450	-4.463253	-0.776748
1	-2.334527	-4.183650	1.497965
6	-2.597106	-2.540625	-1.459790
6	-1.913753	-1.376865	-1.127912
6	1.466319	-1.205144	1.330965
6	2.907597	-0.801119	-0.069566
6	3.060902	0.605303	-0.058707
6	1.437021	1.082683	1.588304
1	-3.015135	-2.653370	-2.453635
1	-1.810276	-0.584024	-1.859463
6	2.212962	0.520623	2.624838
6	2.208625	-0.860948	2.496283
6	2.453363	1.153401	-1.288141
6	2.214721	-1.162454	-1.357947
7	1.906440	0.052260	-1.967729
8	2.399047	2.300124	-1.681187
8	1.960805	-2.259289	-1.794253
1	1.404621	0.128599	-2.840001
1	3.651982	-1.497901	0.286928
1	3.835611	1.163301	0.440915
1	2.794111	1.088949	3.338888
1	2.797320	-1.554320	3.082084
1	1.195335	2.130751	1.499642
1	1.169880	-2.212709	1.078506

RETRO-DA (MIN) (geom. optimized B3LYP/6-311G(d,p)):

6	-3.237626	3.731159	-0.083780
6	-3.676134	2.703192	0.749905
6	-2.982556	1.498632	0.802227
6	-1.825068	1.300597	0.034953
6	-1.404359	2.337276	-0.808756
6	-2.101970	3.541999	-0.866791
6	-1.083877	0.005705	0.117405
6	-1.891862	-1.241400	-0.038822
6	0.236694	-0.037746	0.339748
6	-1.816562	-2.283475	0.895257
6	-2.571596	-3.443439	0.734324
6	-3.418592	-3.581081	-0.362288
6	-3.514059	-2.545993	-1.291627
6	-2.765666	-1.385099	-1.126779
6	1.225401	-1.201587	0.390151
6	2.197768	-0.726330	-0.761283
6	2.229234	0.814385	-0.587712
6	1.259906	1.058070	0.634576
6	1.924524	0.442640	1.862022
6	1.906913	-0.887324	1.717142
6	3.692832	1.164140	-0.346893
6	3.645852	-1.179160	-0.619551
7	4.408893	-0.029879	-0.415275
8	4.172538	2.249768	-0.135800
8	4.082467	-2.301732	-0.664787
1	-4.562175	2.839639	1.359966
1	-3.333474	0.701846	1.448050
1	-0.535594	2.190275	-1.439920
1	-3.781508	4.667881	-0.128245
1	-1.760844	4.329061	-1.530141
1	-1.176615	-2.172081	1.763017
1	-4.006275	-4.483324	-0.488318
1	-2.503015	-4.235928	1.471334
1	-4.175022	-2.642603	-2.145849
1	-2.851950	-0.580741	-1.848592
1	5.408559	-0.067589	-0.262571
1	1.812524	-1.053053	-1.728483
1	1.876058	1.362483	-1.462751
1	2.391642	1.014542	2.652864
1	2.359484	-1.627080	2.364256
1	0.910802	2.080926	0.731579
1	0.849934	-2.210351	0.254852

REFERENCES:

[S1] Nucci, M.; Marazzi, M.; Frutos, L. M. Mechanochemical Improvement of Norbornadiene-Based Molecular Solar–Thermal Systems Performance. *ACS Sustain. Chem. Eng.* **2019**, *7*, 19496–19504. https://doi.org/10.1021/acssuschemeng.9b04503.

[S2] Pladevall, B. S.; de Aguirre, A.; Maseras, F. Understanding Ball Milling Mechanochemical Processes with DFT Calculations and Microkinetic Modeling. *ChemSusChem*, **2021**, *14*, 2763–2768. https://doi.org/10.1002/cssc.202100497.
